# Real-time liver tracking algorithm based on LSTM and SVR networks for use in surface-guided radiation therapy

**DOI:** 10.1186/s13014-020-01729-7

**Published:** 2021-01-14

**Authors:** Guangyu Wang, Zhibin Li, Guangjun Li, Guyu Dai, Qing Xiao, Long Bai, Yisong He, Yaxin Liu, Sen Bai

**Affiliations:** 1 Department of Radiation Oncology, Cancer Center and State Key Laboratory of Biotherapy, West China Hospital, Sichuan University, Chengdu, China; 2 College of Physics, Sichuan University, Chengdu, 610065 China

**Keywords:** Radiotherapy, Respiratory motion, Liver tracking, LSTM, SVR, Prediction

## Abstract

**Background:**

Surface-guided radiation therapy can be used to continuously monitor a patient’s surface motions during radiotherapy by a non-irradiating, noninvasive optical surface imaging technique. In this study, machine learning methods were applied to predict external respiratory motion signals and predict internal liver motion in this therapeutic context.

**Methods:**

Seven groups of interrelated external/internal respiratory liver motion samples lasting from 5 to 6 min collected simultaneously were used as a dataset, D_v_. Long short-term memory (LSTM) and support vector regression (SVR) networks were then used to establish external respiratory signal prediction models (LSTMpred/SVRpred) and external/internal respiratory motion correlation models (LSTMcorr/SVRcorr). These external prediction and external/internal correlation models were then combined into an integrated model. Finally, the LSTMcorr model was used to perform five groups of model updating experiments to confirm the necessity of continuously updating the external/internal correlation model. The root-mean-square error (RMSE), mean absolute error (MAE), and maximum absolute error (MAX_AE) were used to evaluate the performance of each model.

**Results:**

The models established using the LSTM neural network performed better than those established using the SVR network in the tasks of predicting external respiratory signals for latency-compensation (RMSE < 0.5 mm at a latency of 450 ms) and predicting internal liver motion using external signals (RMSE < 0.6 mm). The prediction errors of the integrated model (RMSE ≤ 1.0 mm) were slightly higher than those of the external prediction and external/internal correlation models. The RMSE/MAE of the fifth model update was approximately ten times smaller than that of the first model update.

**Conclusions:**

The LSTM networks outperform SVR networks at predicting external respiratory signals and internal liver motion because of LSTM’s strong ability to deal with time-dependencies. The LSTM-based integrated model performs well at predicting liver motion from external respiratory signals with system latencies of up to 450 ms. It is necessary to update the external/internal correlation model continuously.

## Introduction

During the radiotherapy of thoracic–abdominal tumors, respiratory motion can cause tumor displacement that affects the accuracy of radiotherapy [1]. In particular, stereotactic body radiation therapy (SBRT) technique [2] has increased requirements in terms of irradiation accuracy and, therefore, will be impacted more by respiratory motion. To manage the tumor motion caused by respiration, motion-encompassing [3, 4] and breath-holding methods [5–8] as well as forced shallow-respiratory with abdominal compression [9], respiratory-gating [10, 11], and respiration-synchronized techniques [12] are used clinically. Both respiratory-gating and respiration-synchronized techniques require the real-time tracking of tumors. Tumor tracking techniques can be divided into two categories. The first is direct real-time tracking technique, in which X-ray imaging is used to locate tumors or implanted metal markers, or electromagnetic methods are used to track implanted coils in the target volume. These methods either require additional irradiation or are invasive [13–16]. The second approach involves the indirect real-time tracking technique, in which tumor position is predicted based on external surrogate respiratory signals acquired using optical or infrared devices [17, 18] or spirometry [19]. Owing to its non-radiological and non-invasive nature, the indirect real-time tracking technique can be applied in the clinic using surface-guided radiation therapy (SGRT). This technique has many advantages such as imaging without the requirement of dosing, real-time feedback, 3D sub-millimeter spatial resolution, non-invasive and non-contact application, ease of use, and an enhanced field of view (FOV) [20]. These advantages make SGRT well-suited to real-time tracking and respiratory gating, and many studies have reported applications of SGRT in the treatment of lung [21] and breast tumors [22], laryngeal cancer [23], etc.

Establishing an accurate external respiratory signal prediction model and an appropriate external/internal correlation model between external surrogates and internal tumor motion is the key to the successful implementation of indirect real-time tumor tracking technology [24]. The external respiratory signal prediction and external/internal respiratory motion correlation models allow for the compensation of system latency and the prediction of internal tumor motion, respectively. In respiratory gating in clinical settings, the latency of SGRT can result in treatment inefficiencies and/or geographic misses [25]. According to the AAPM TG 142 report [26], latency should be within 100 ms, a tolerance that assumes a moving object traveling at a speed that is not higher than 20 mm/s, corresponding to a positional uncertainty of 2 mm [26]. However, the total latency measured in clinical practice is often greater than 100 ms, and cases involving latencies of 200–400 ms are often reported [27, 28]. In such cases, the positional uncertainty of the tumor can reach more than 8 mm, which can cause unexpected loss of target dose coverage and result in cold spots [29]. Moreover, the positional uncertainty caused by high latency can also lead to interplay effects, blurring and spatial deformation of the dose distribution [30], and substantial discrepancies between planned and delivered doses [31]. The system latency is inherent in a real-time tracking system associated with the processes of obtaining external imaging data, predicting internal tumor position from the external/internal correlation model, and adjusting the radiation beam accordingly [32]. Different studies have shown that the system latencies of different real-time tracking devices range from tens of milliseconds to more than 400 ms [32–34]. As mentioned previously, a latency of 100 ms will result in a tumor positional uncertainty of 2 mm [26].

External respiratory motion prediction algorithms developed in previous studies can be divided into two categories. The first include algorithms based on existing models, primarily the least-squares fitting algorithm [35], algorithms based on the extended Kalman filter [36], and fuzzy logic algorithms [17, 37]. Such algorithms are based on the assumption that the existing respiratory pattern remains stationary and periodic—an assumption that might be incorrect [24]. The second category concerns model-free prediction algorithms, including neural networks [38–40], regression analysis [41], and support vector regression (SVR) [42]. The advantage of these algorithms is that they do not require a stable respiratory pattern.

External/internal correlation algorithms developed in other studies primarily apply linear [43, 44], piecewise linear [45], polynomial [46], and combined polynomial [47] models or adaptive filters, neural networks [48, 49], or SVR [50]. However, relatively simple linear polynomial models suffer from poor robustness and produce relatively large prediction errors for samples with large volatility [46]. Algorithms such as filters, neural networks such as long short-term memory (LSTM), and SVR networks are stronger at processing nonlinear problems and have better real-time dynamic prediction performance. The LSTM neural network was first proposed by Hochreiter and Schmidhuber [51] in 1997. It has been recognized for its outstanding performance in processing sequence information and is widely used in speech recognition and machine translation tasks [52, 53]. SVR is an important application branch of support vector machines (SVMs) that is used to minimize structural risks and seek globally optimal solutions. SVR applies nonlinear transformation to convert actual nonlinear problems into high-dimensional feature spaces in which the nonlinear problems can be solved by constructing linear decision functions [54]. Based on the characteristics and advantages of LSTM and SVR mentioned above, the LSTM and SVR networks were used to establish an external/internal respiratory motion correlation model (external/internal correlation model) and an external respiratory signals prediction model (external prediction model) in this study.

Indirect real-time tracking techniques based on external surrogate respiratory signals are completely free of additional radiation and are noninvasive. To implement these techniques, an external prediction model must be combined with an external/internal correlation model. All of the previous studies in this area were limited to external respiratory motion or internal tumor/organ motion prediction alone and did not reflect actual clinical situations, in which latency compensation and the prediction of internal tumor position should be performed simultaneously. Considering the limitation of the previous studies above, these models were combined into an integrated model for clinical practice in this study. The integrated model uses external respiratory motion signals to predict internal liver motion and compensates for system latency. The performance of the respective networks and their combination was compared and analyzed. Finally, to reflect the temporally changing relationship between external and internal respiratory motion [55, 56], the necessity of continuously updating the model was also verified.

## Methods

### Dataset

The respiratory motion data of seven volunteers collected by the Institute of Robotics and Cognitive Systems of the University of Lübeck in Germany [47, 57] were used. The respiratory motion signals of the chest surface and internal liver were collected in the x- (left–right), y- (superior-inferior), and z- (anterior–posterior) directions, respectively. The samples collected for each volunteer were taken as individual sets of data, which were combined to form the final dataset, D_v_. The respiratory motion signals of the internal liver were obtained using 4D ultrasound and template matching to track the motion of the liver vessel bifurcation point. In the template matching process, phase-only correlation (POC) [58] was performed followed by normalized cross-correlation (NCC) [59] with interpolation on the target volume to determine the point of optimal registration; details on this process can be found in [47]. External respiratory motion samples were obtained using an AccuTrack 250 system to track the motion of LEDs fixed on the surface of each volunteer’s chest [47]. The duration of respiratory motion sample collection for each set was 5–6 min, and approximately 6500 to 8100 sampling points were obtained in each direction per set. The sampling frequency of the internal respiratory motion sample was 20 Hz. Each external respiratory motion sample was resampled to make it consistent in length with the corresponding internal respiratory motion sample. Prior to model training, preprocessing operations such as outlier deletion, smoothing, filtering, and standardization were performed on D_v_.

### LSTM network and SVR network

#### LSTM network

Sets of training and training label samples (X_train_ = [x_1_, x_2_, …, x_m_] and Y_train_ = [y_1_, y_2_, …, y_m_], respectively) and testing and testing label samples (X_test_ = [x_m+1_, x _m+2_, …, x_m+n_] and Y_test_ = [y_m+1_, y_m+2_, …, y_m+n_], respectively) were obtained for the LSTM network. The LSTM network comprises multiple memory blocks, each of which contains one cell and three gates (input, forget, and output gates) [60]. Through a forward propagation process, the LSTM network turns input data into output samples. The entire forward propagation of one block from input to output is carried out as follows:

The output $b_{l}^{t}$ of the input gate is

1 $$b_{l}^{t} = f\left( {a_{l}^{t} } \right),$$

where

2 $$a_{l}^{t} = \mathop \sum \limits_{i = 1}^{I} w_{il} x_{i}^{t} + \mathop \sum \limits_{c = 1}^{C} w_{cl} s_{c}^{t - 1} + \mathop \sum \limits_{h = 1}^{H} w_{hl} b_{h}^{t - 1} .$$

The output $b_{\phi }^{t}$ of the forget gate is

3 $$b_{\phi }^{t} = f\left( {a_{\phi }^{t} } \right),$$

where

4 $$a_{\phi }^{t} = \mathop \sum \limits_{i = 1}^{I} w_{i\phi } x_{i}^{t} + \mathop \sum \limits_{c = 1}^{C} w_{c\phi } s_{c}^{t - 1} + \mathop \sum \limits_{h = 1}^{H} w_{h\phi } b_{h}^{t - 1} .$$

The output $b_{w}^{t}$ of the output gate is

5 $$b_{w}^{t} = f\left( {a_{w}^{t} } \right),$$

where

6 $$a_{w}^{t} = \mathop \sum \limits_{i = 1}^{I} w_{iw} x_{i}^{t} + \mathop \sum \limits_{c = 1}^{C} w_{cw} s_{c}^{t} + \mathop \sum \limits_{h = 1}^{H} w_{hw} b_{h}^{t - 1} .$$

From these outputs, the output $s_{c}^{t}$ of the cell is obtained as

7 $$S_{c}^{t} = b_{\phi }^{t} s_{c}^{t - 1} + b_{l}^{t} g(a_{c}^{t} ),$$

where

8 $$a_{c}^{t} = \mathop \sum \limits_{i = 1}^{I} w_{ic} x_{i}^{t} + \mathop \sum \limits_{h = 1}^{H} w_{hc} b_{h}^{t - 1} .$$

the final output of the block, $b_{c}^{t}$, is then calculated as the product of $b_{w}^{t}$ and a function of $s_{c}^{t}$:

9 $$b_{c}^{t} = b_{w}^{t} h(s_{c}^{t} ).$$

It should be noted that, in the above equations, f, g, and h denote the activation functions used by the three gates, the input, and the final output, respectively.

#### SVR network

Sets of training and testing samples (D_train_ = {(x_1_,y_1_), (x_2_,y_2_), …, (x_m_,y_m_), yi $\in$ R} and D_test_ = {(x_m+1_,y _m+1_), (x _m+2_,y _m+2_), …, (x_m+n_,y_m+n_), yi $\in$ R}, respectively) were obtained for the SVR network, which attempts to find a model f(x) in which y* = f(x_i_) and y_i_ are as close as possible [54, 61]. For a maximum tolerable deviation between y* and y_i_ of ϵ, the SVR problem can be formalized as

10 $$\mathop {min}\limits_{w,b} \frac{1}{2} \| w \|^{2} + C\mathop \sum \limits_{i = 1}^{m} l_{\epsilon} \left( {f\left( {x_{i} } \right),y_{i} } \right),$$

where C is the regularization constant and $l_{\epsilon }$ is the ϵ-insensitive loss function.

By introducing the slack variables $\xi_{i}$ and $\hat{\xi }_{i}$, Eq. (10) can be expressed as

11 $$\mathop {min}\limits_{{w,b,\xi_{i} ,\hat{\xi }_{i} }} \frac{1}{2}\|w\|^{2} + C \mathop \sum \limits_{i = 1}^{m} (\xi_{i} ,\hat{\xi }_{i} ).$$

The Lagrange multiplier $u_{i}$ can then be introduced to obtain the SVR solution as

12 $$f\left( x \right) = \mathop \sum \limits_{i = 1}^{m} (\hat{\alpha }_{i} - \alpha_{i} )x_{i}^{T} x + b,$$

where b is the model parameter to be determined and f(x) is the final model found by the SVR method.

### External respiratory signals prediction models

The LSTM prediction (LSTMpred) and SVR prediction (SVRpred) models were established using the LSTM neural and SVR networks, respectively, to predict the respiratory motion of the chest surface at the system latency. Both LSTMpred and SVRpred use the current external respiratory motion signals S_t_ to predict the future external respiratory motion signals S_t+i_. Considering that the length of the training and label datasets should be the same during the training of the SVR model, a S_t_-to-S_t+i_ length ratio of 1:1 was selected for both models. As the typical system latency ranges from tens of milliseconds to more than 400 ms, the respiratory motion prediction algorithm results were assessed at latencies of 50, 150, 200, and 450 ms, i.e., the i = 1, 3, 4, and 9 latencies were set to 50, 150, 200, and 450 ms, respectively.

**Fig. 1:**
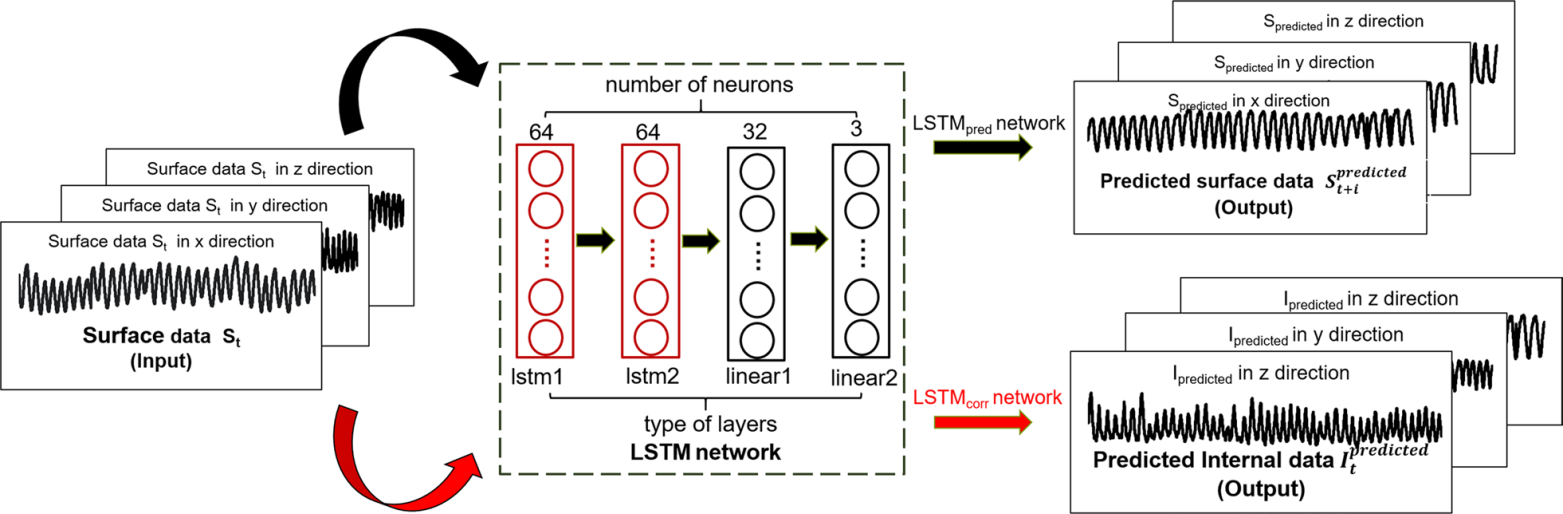
Structure of LSTMpred and LSTMcorr models

The external respiratory motion data of dataset D_v_ were divided at ratios of 9:1 into training and testing data for the LSTMpred and SVRpred models. After testing, comparison, and adjustment to determine the best network performance, appropriate parameters were selected for the two external prediction models. For the LSTMpred model, mean square error (MSE) loss was chosen as the loss function and the network was trained using an Adam optimizer with a learning rate of 0.001 and a batch size of 64. A dropout rate of 20% over 60 total epochs and 20 time steps was used in the LSTMpred model adjustment process. The number of layers and neurons in each layer are shown in Fig. 1. For the SVRpred model, the radial basis function (RBF) kernel was selected as the kernel function, and the gamma and penalty parameter C of SVRpred were set as 0.1 and 1000, respectively.

### External/internal respiratory motion correlation models

The LSTM and SVR networks were used to establish the external/internal correlation models LSTMcorr and SVRcorr, respectively, which use the current external respiratory motion signal S_t_ to predict the current internal liver respiratory motion sample I_t_. The preprocessed dataset D_v_ was divided at 9:1 ratios into training and testing data for the LSTMcorr and SVRcorr models. Because the external and internal samples had the same length and corresponded to each other, a S_t_-to-I_t_ ratio of 1:1 was selected for both models.

The structure and parameter selection processes applied in obtaining the external/internal correlation models were equivalent to those used for the external prediction models except that 40 instead of 60 epochs were used to train LSTMcorr. The external/internal correlation and external prediction models differ primarily in terms of the training and label data used to train the respective models. Both sets of models were developed based on a Pytorch deep learning framework using Python.

### Integrated model

The integrated model derived as a combination of the trained external prediction and trained external/internal correlation models uses external respiratory motion signals to predict internal liver motion while compensating for system latency. The efficiency of this combination of the two models was assessed by predicting future internal liver positions.

### Verification of external/internal correlation model update

During free breathing, different parts of the anatomy can move with different temporal and spatial relationships that will change continuously over time [24, 62]. Unexpected behaviors such as talking and coughing can also induce obvious changes in these relationships. Therefore, the established external/internal correlation model should also change over time. Obtaining the latest sample and updating the model continuously is a common approach to solving this problem [24]. In general, the changes in the temporal and spatial relationships will be slow, and approximately 30 s will be required to detect obvious alterations [48]. Previous studies have shown that updating every 10 s is sufficient to ensure the accuracy of a model [43]. In the model update process, the weights of each computing node within the neural network are updated automatically by entering the latest collected data as input to the network, which does not change the structure of the network or the set parameters.

Although the changing characteristics of a patient’s respiratory motion pattern over time will also affect the prediction of external respiratory motion, the influence of this is primarily manifested on a time scale that is longer than the system latency. Thus, the update verification of the model primarily focuses on the external/internal correlation.

To carry out update verification of the LSTMcorr model, the dataset D_v_ was divided into six parts—C1, C2, C3, C4, C5, and C6—in the proportion 1:2:2:2:2:1. Five groups of assessments were carried out using C1, C1 + C2, C1 + C2 + C3, C1 + C2 + C3 + C4, and C1 + C2 + C3 + C4 + C5 as the training sets and the unified C6 as the testing set. Each group of assessments was carried out five times. The $\overline{RMSE} /\overline{MAE}$ of each assessment was taken as the final result for comparison, with the results for each of the five groups normalized to the fifth update to enable an intuitive comparison.

### Evaluation

The root-mean-squared errors (RMSEs), mean absolute errors (MAEs), and maximum absolute errors (MAX_AEs) of the external prediction, external/internal correlation, and integrated models were used as evaluation indicators to assess the deviations between the predicted and true results, $\hat{y}_{i}$ and $y_{i}$, respectively. These indicators are defined as follows:

13 $$RMSE = \sqrt {\frac{1}{N}\mathop \sum \limits_{i = 1}^{N} (y_{i} - \hat{y}_{i} )^{2} } ,$$

14 $$MAE = \frac{1}{N}\mathop \sum \limits_{i = 1}^{N} \left| {y_{i} - \hat{y}_{i} } \right|,$$

15 $${\text{MAX}}\_{\text{AE}} = \max \left( {\left| {y_{i} - \hat{y}_{i} } \right|} \right).$$

In each case, a smaller indicator value corresponds to a predicted result that is closer to the real result and a better-performing model.

## Results

### External respiratory motion prediction

**Fig. 2:**
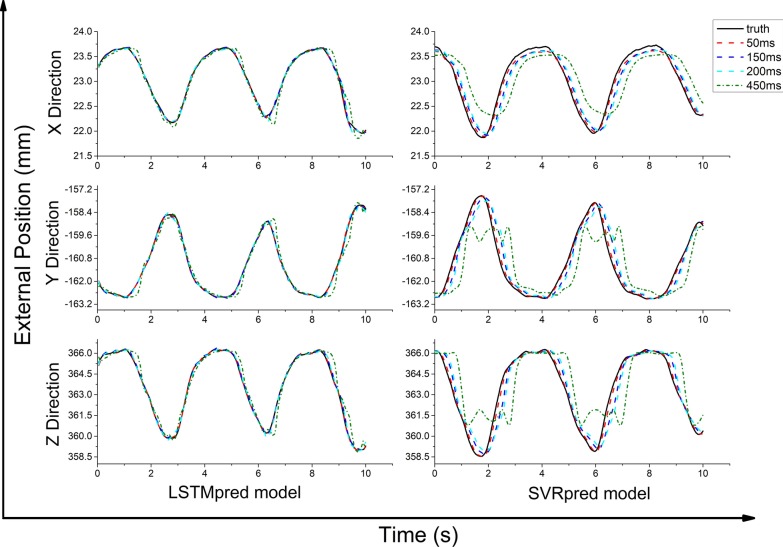
Change in difference in three directions between predicted position (mm) obtained from LSTMpred and SVRpred models and real position (mm) from external samples as a function of latency

**Fig. 3:**
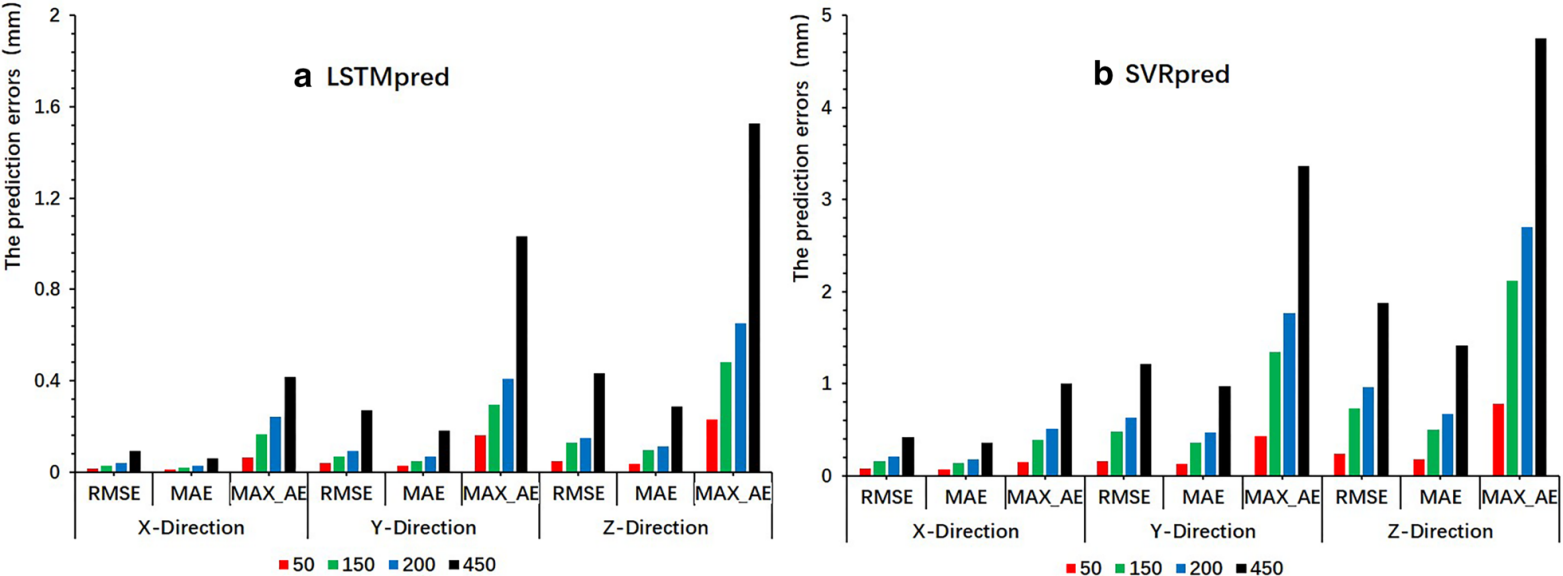
RMSE, MAE, and MAX_AE of **a** LSTMpred and **b** SVRpred models in three directions at different latencies

The LSTMpred model was found to be much more accurate in all directions than the SVRpred model as an external prediction model (see Figs. 2, 3). For the LSTMpred model with a latency of 450 ms, the maximum prediction errors for all tested cases in the x-, y-, and z-directions were 0.415, 1.034, and 1.529 mm, respectively, while those for the SVRpred model were 1.001, 3.368, and 4.749 mm, respectively. The prediction errors of the LSTMpred and SVRpred models were both largest in the z-direction, followed by the y- and x-directions. The RMSE, MAE, and MAX_AE values of both models both increased with the latency, indicating that increasing the latency increased the prediction error. This occurred because the human respiratory pattern changes over time and, therefore, increasing the system latency increases the difference between the predicted and current external respiratory motion signals.

### External/internal correlation model

**Fig. 4:**
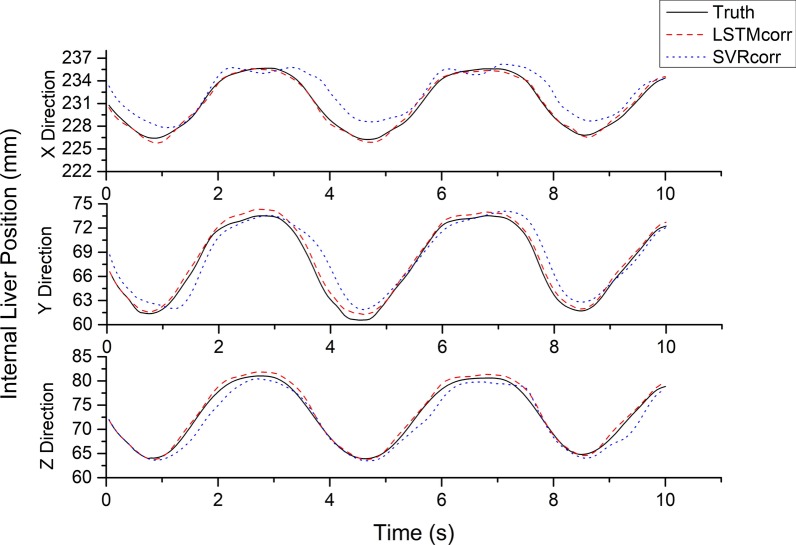
Predicted positions (mm) obtained from LSTMcorr and SVRcorr models and actual positions (mm) of liver in three directions

**Fig. 5:**
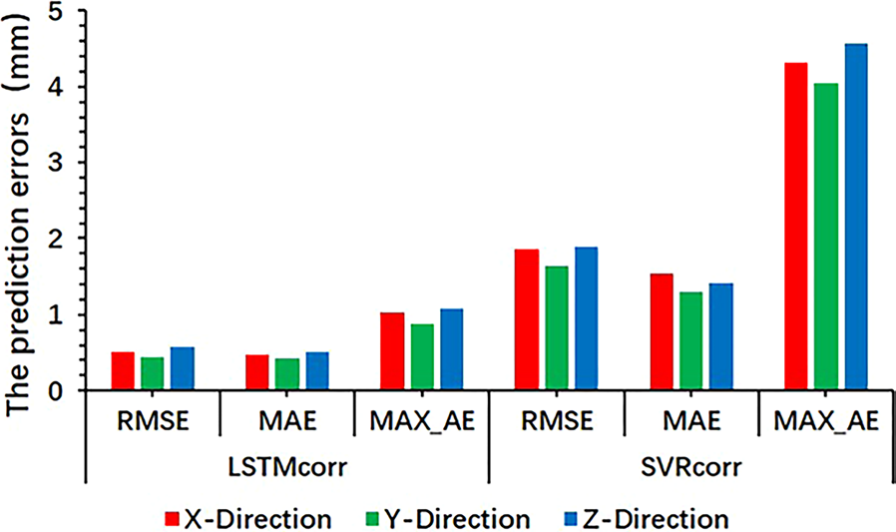
RMSE, MAE, and MAX_AE of LSTMcorr and SVRcorr models in three directions

The LSTMcorr model was found to be much more accurate in all directions than the SVRcorr model as an external/internal correlation model (see Figs. 4, 5). The RMSE, MAE, and MAX_AE values of the LSTMcorr model were much smaller than those of the SVRcorr model in all directions, with maximum prediction errors for all tested cases of 1.027, 0.886, and 1.081 mm in the x, y, and z directions, respectively.

### Integrated model

**Fig. 6:**
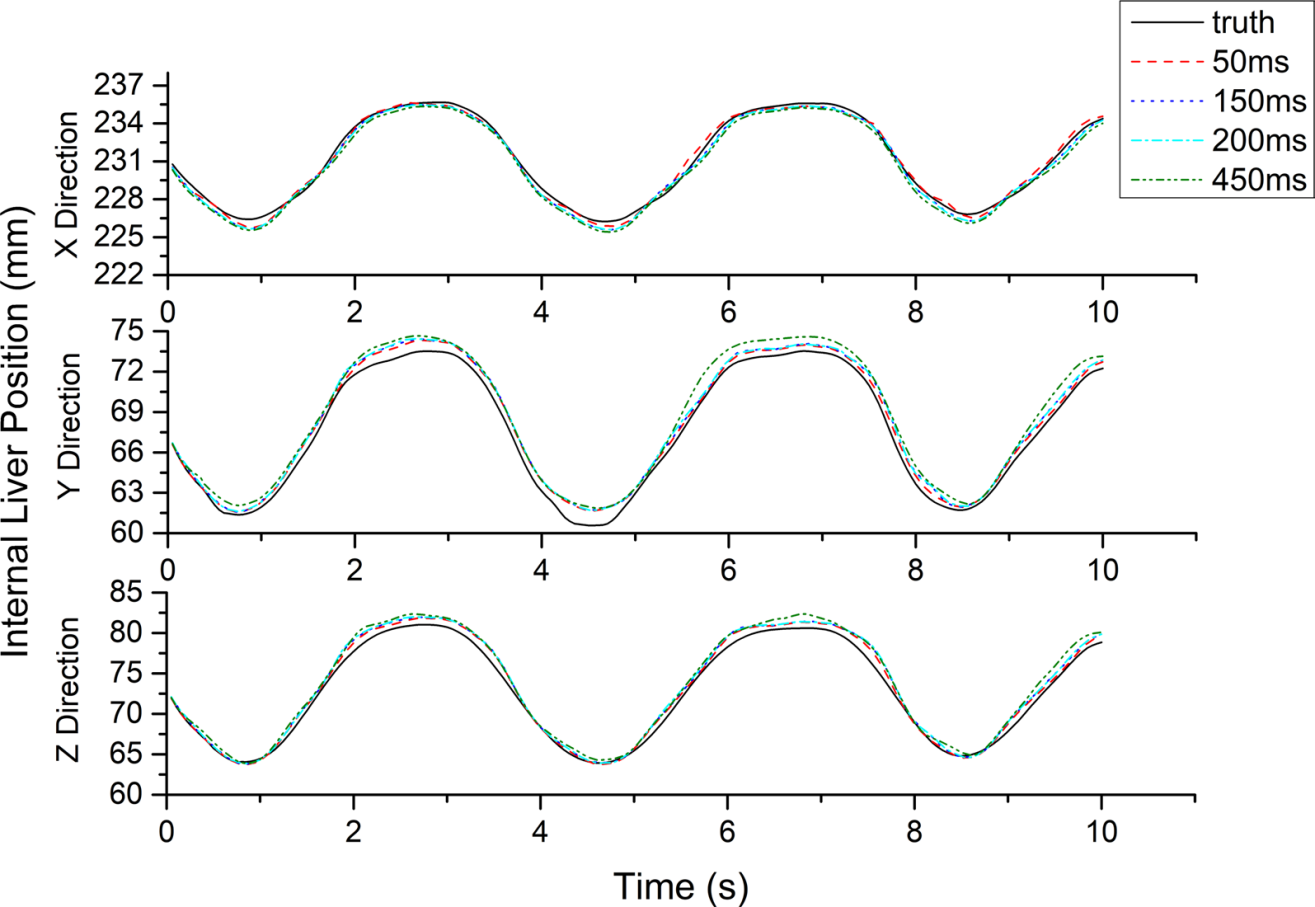
Predicted position (mm) in three directions obtained from integrated model and real position (mm) of liver at different latencies

**Fig. 7:**
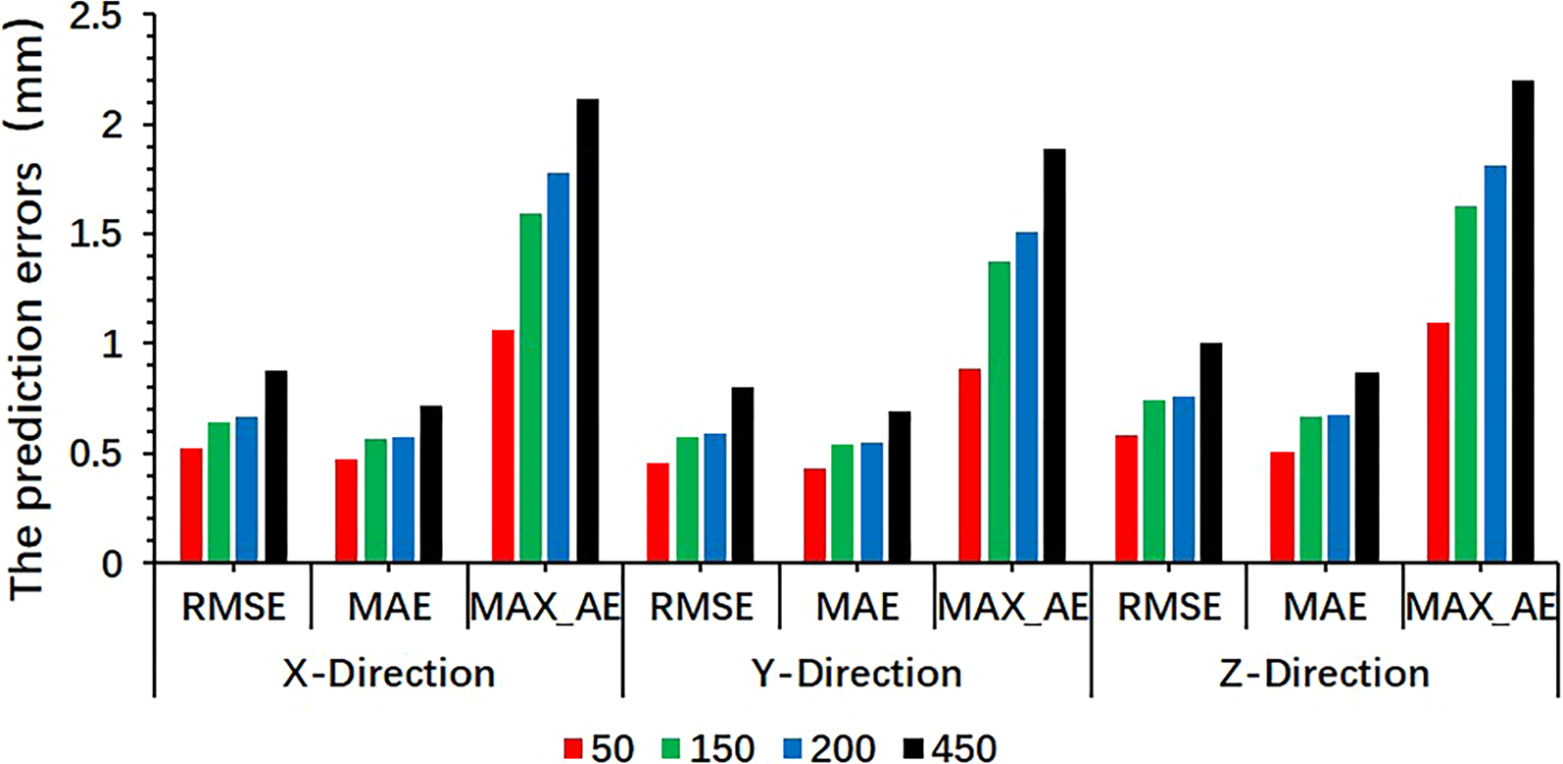
RMSE, MAE, and MAX_AE of the integrated model at different latencies in three directions

Because the LSTMpred and LSTMcorr external and external/internal correlation models had smaller errors than the SVRpred and SVRcorr prediction models, the integrated model was constructed using the LSTMpred and LSTMcorr models. The RMSE, MAE, and MAX_AE of the integrated model were all found to increase with the latency in all directions (see Figs. 6, 7), with RMSE and MAX_AE values for all tested cases of approximately 1 and 2 mm, respectively. In addition, the prediction errors of the integrated model were slightly greater than those of the external prediction and external/internal correlation models but smaller than the sum of their errors.

### Verification for external/internal correlation model update

**Fig. 8:**
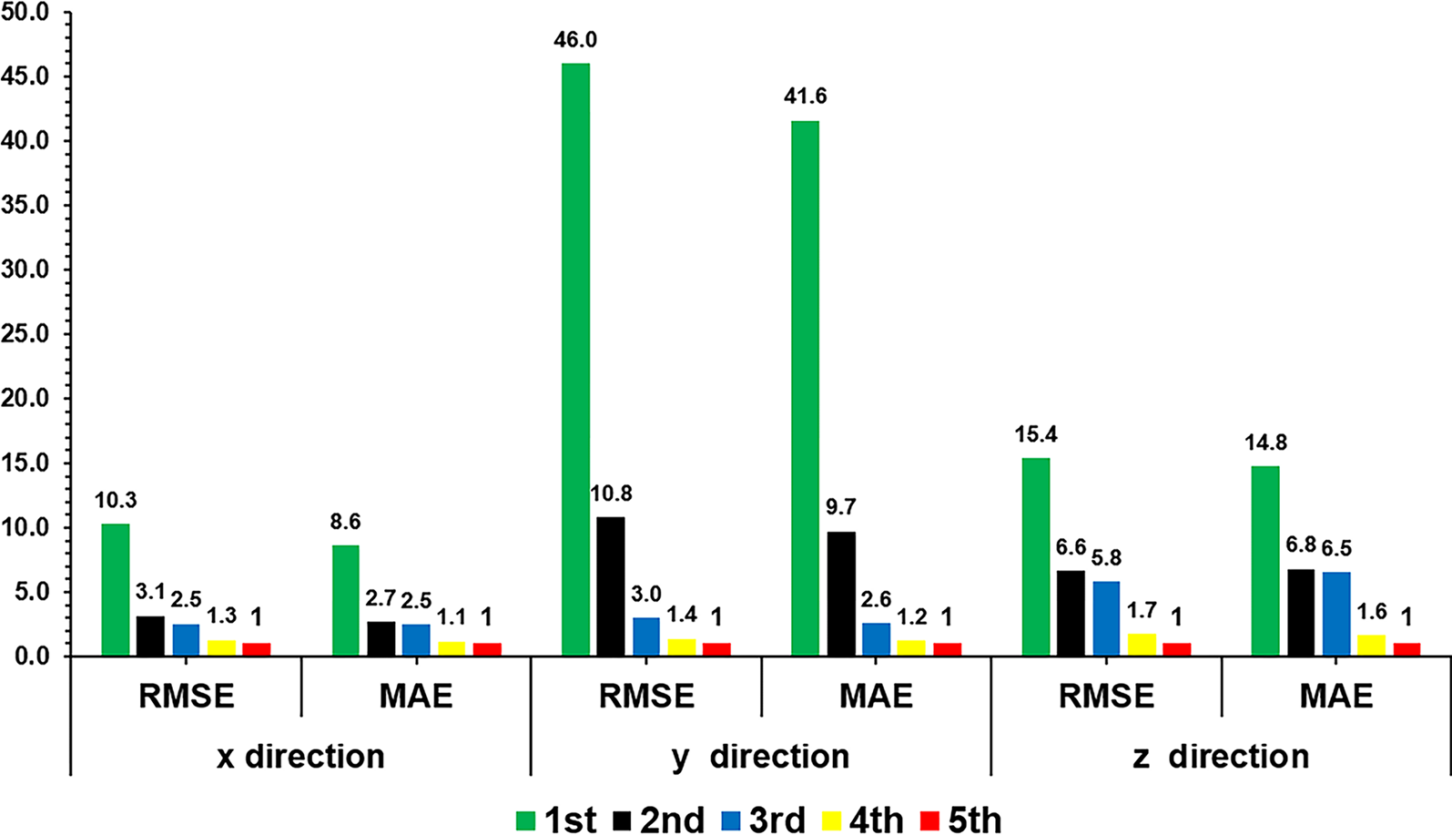
RMSE and MAE of first to fifth external/internal correlation model updates (the RMSE and MAE are all normalized to the fifth update)

As the external/internal correlation model is continuously updated, its RMSE and MAE are gradually reduced in all directions (see Fig. 8). As the time interval between the training and test samples decreases, the magnitude of error reduction becomes progressively smaller because reducing the time interval between the two reduces the difference in the respiratory motion pattern between the training samples and the test data.

## Discussion

The external respiratory motion prediction and external/internal correlation models for predicting internal liver motion established by the LSTM neural network universally outperformed those established by the SVR network in all directions. The primary reason for this is that the LSTM neural network can effectively solve time-dependency problems [63, 64] as a result of the network’s hidden unit, which controls memory and forgetting of time-series information to enable the selective remembering and transmission of these data [65]. In addition, the nonlinear fitting ability of the SVR network is based on the use of similar history samples, and the SVR cannot reflect further abnormal fluctuations using rare similar history samples [66]. By contrast, LSTM networks have stronger feature extraction capabilities than SVR networks [67–69] and can use these to learn additional features pertaining to abnormal fluctuations. These features provide LSTM networks with more powerful processing capabilities for abnormal fluctuations and gives them smaller prediction errors than SVR networks. Studies in a number of fields, including stock premium [70], snowmelt driven stream flow [71], and traffic flow prediction [69] have shown the superiority of LSTM networks relative to SVR networks in processing time-series data.

In this study, we established an integrated model that predicts internal liver motion from external respiratory signals based on actual clinical tumor real-time motion tracking scenarios and took system latency into account in developing the model. The maximum RMSE and MAX_AE of the integrated model are approximately 1 and 2 mm, respectively, in all directions, a degree of accuracy that can meet clinical requirements for the real-time motion tracking of liver tumors [72].

A patient’s respiratory pattern will change over time as a result of the complex patterns of simultaneous motion of different anatomical structures, which produce phase difference variation and time-changing spatiotemporal correlations [73]. Despite this accepted fact, to date, there have been few relevant external/internal correlation experiments to verify the necessity of model updating in radiotherapy-based tumor real-time tracking; furthermore, all such studies have been limited to exploring the impact of short-term updates [43, 55]. The results of our model update assessments indicate that the RMSE/MAE obtained for the fifth model update is smaller than that obtained for the first update by an approximate factor of ten in a long-term state. Because the RMSE/MAE decreases as the model is updated, it is necessary to continuously update the model. Doing so ensures that the respiratory motion correlation established by the model matches the situation during the current moment, thereby reducing prediction errors, improving prediction accuracy, and reducing the large impact of random patient actions. Furthermore, the accuracy of the fifth model update is close to that of the fourth update, indicating that when the training/prediction sample length ratio reaches 7:3, a high degree of accuracy can be achieved in the external/internal correlation model.

This study had several limitations. First, the networks employed in the study were specific in that they were all trained based on a respiratory motion dataset obtained from a single institution. Before applying the network developed in this study to any other dataset, therefore, the models’ structure and parameters will have to be adjusted and the models will have to be retrained and reevaluated. In addition, the models’ performance could be improved by using a larger dataset or a dataset containing multicenter data. Second, the model for predicting internal liver motion from external respiratory signals developed in this study is only suitable when exposed surface skin immobilization devices such as vacuum bags and stereotactic body frames are used. The model cannot be applied when thermoplastic frame immobilization devices are used because it would not be able to accurately obtain external motion signals and changes in the patient's breathing motion pattern. Third, we only tracked the motion of the liver vessel bifurcation point in our model and, therefore, did not consider nonrigid motion such as deformation of the liver. Finally, a patient’s respiratory motion can be severely affected by many factors, including initial nervousness or activity during setup and commencement of treatment followed by eventual relaxation on the table, which alter their breathing pattern. As a result, the application of motion compensation might not always be appropriate, and the question of whether certain patients are suitable for motion compensation treatment alone will have to be explored [74]. In future studies, we will further explore and evaluate the benefits of motion compensation for specific patients.

## Conclusion

In this study, it was shown that LSTM networks outperform SVR networks in the prediction of external respiratory signals and internal liver motion because of the strong ability of the former to capture time dependencies. An LSTM-based integrated model was found to perform well in predicting liver motion from external respiratory signals with a system latency of up to 450 ms. It was also confirmed that continuously updating the model is necessary to improve its prediction accuracy. Our study, could be a meaningful contribution to the real-time motion tracking of liver tumors in clinical practice.

## Data Availability

The link of datasets used during the current study is available from the paper of references 47.

## Abbreviations

FOV: Field of view
LSTM: Long-short term memory
LSTMcorr: The external/internal respiratory motion correlation model established by LSTM network
LSTMpred: The external respiratory signals prediction model established by LSTM network
MAE: Mean absolute errors
MAX_AE: Maximum absolute errors
MSE: Mean square error
NCC: Normalized cross-correlation
POC: Phase-only correlation
RBF: Radial basis function
RMSE: Root mean squared errors
SBRT: Stereotactic body radiation therapy
SGRT: Surface-guided radiation therapy
SVM: Support vector machine
SVR: Support vector regression
SVRcorr: The external/internal respiratory motion correlation model established by SVR network
SVRpred: The external respiratory signals prediction model established by SVR network
